# Genetic incompatibility drives mate choice in a parasitic wasp

**DOI:** 10.1186/1742-9994-10-43

**Published:** 2013-07-30

**Authors:** Andra Thiel, Anne C Weeda, Jetske G de Boer, Thomas S Hoffmeister

**Affiliations:** 1 Population and Evolutionary Ecology, Institute of Ecology, University of Bremen, FB 2, Leobener Str. NW2, 28359 Bremen, Germany; 2 Evolutionary Genetics, Centre for Ecological and Evolutionary Studies, University of Groningen, P.O.Box 11103, 9700, CC Groningen, The Netherlands; 3 Laboratory of Entomology, Wageningen University, P.O. Box 8031, 6700, EH Wageningen, The Netherlands

**Keywords:** Genetic compatibility, Mate choice, Allele recognition, Diploid males, Extinction vortex, Complementary sex determination, Disassortative mating, MHC

## Abstract

**Introduction:**

Allelic incompatibility between individuals of the same species should select for mate choice based on the genetic make-up of both partners at loci that influence offspring fitness. As a consequence, mate choice may be an important driver of allelic diversity. A complementary sex determination (CSD) system is responsible for intraspecific allelic incompatibility in many species of ants, bees, and wasps. CSD may thus favour disassortative mating and in this, resembles the MHC of the vertebrate immune system, or the self-incompatibility (SI) system of higher plants.

**Results:**

Here we show that in the monogamous parasitic wasp *Bracon brevicornis* (Wesmael), females are able to reject partners with incompatible alleles. Forcing females to accept initially rejected partners resulted in sex ratio distortion and partial infertility of offspring.

**Conclusions:**

CSD-disassortative mating occurred independent of kin recognition and inbreeding avoidance in our experiment. The fitness consequences of mate choice are directly observable, not influenced by environmental effects, and more severe than in comparable systems (SI or MHC), on individuals as well as at the population level. Our results thus demonstrate the strong potential of female mate choice for maintaining high offspring fitness in this species.

## Introduction

Mate choice, the non-random selection of mates, is extremely widespread in sexually reproducing animals. Sometimes, direct benefits such as resources are accrued, but often, females ‘shop’ for genetic benefits to increase the fitness of their offspring [1]. Mate choice may then be based on ‘good genes’ indicating the heritable quality of mates, or on ‘genetic compatibility’ [2,3]. The latter concept implies that benefits gained by optimal mate choice are conditional on the genetic makeup of both partners at loci that influence offspring fitness. Some of the best-studied examples of mate choice based on genetic compatibility concern the major histocompatibility complex (MHC) in vertebrates. Diversity at MHC loci can enhance immunity against parasites and pathogens (e.g. [4]) and a preference for MHC-dissimilar mates exists in a variety of animals such as fish, birds, and mammals including humans (e.g. [5-7]). Mate choice is discussed as an important driver of allelic diversity within species [2,7], even though the evidence is often inconsistent across studies [6-10]. That might be because the fitness benefits of MHC-disassortative mating depend on environmental factors, e.g. the exposure to parasites, and may thus vary between species and with experimental conditions [11,12].

**Figure 1:**
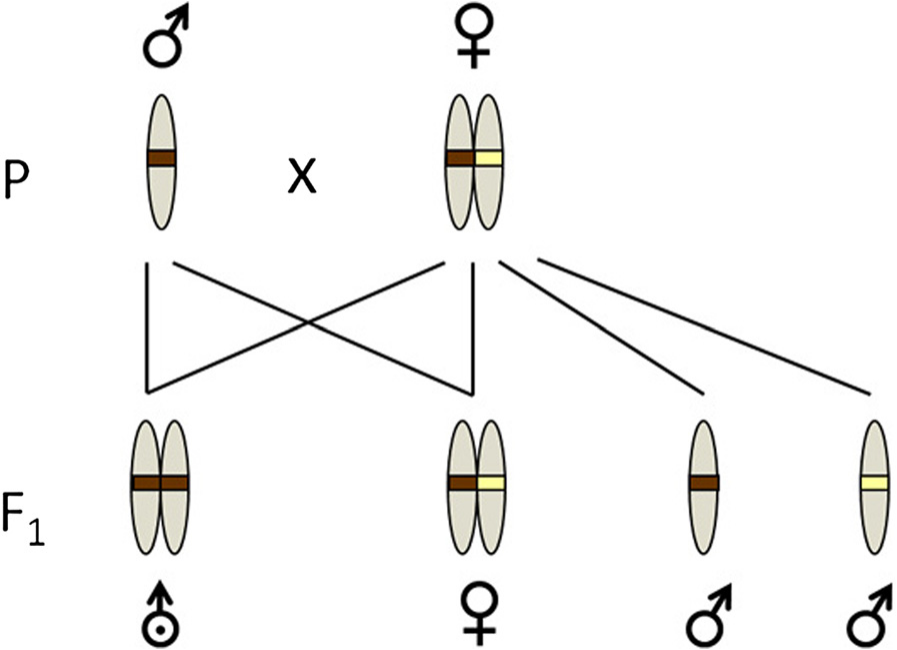
***Bracon brevicornis *reproductive biology.** Matched matings in parental generation P result in three kinds of offspring: diploid homozygous males (F1 on the left; identical colour bands on chromosomes), diploid heterozygous females (F1 centre; different colour bands), or haploid hemizygous males, from unfertilized eggs (F1 on the right).

While genetic compatibility may enhance fitness in vertebrates, it is essential for offspring development in plants with a self-incompatibility (SI) system [13]. Genetic compatibility is also critical in hymenopteran insects exhibiting either single-locus or multiple-locus complementary sex determination (sl-CSD or ml-CSD, respectively). In those species, heterozygosity at the sex locus (or loci) is directly linked to offspring fitness because it initiates the pathway to female development in fertilized, diploid eggs [14]: Sons normally develop from unfertilized eggs and are haploid. However, diploid males are produced when eggs are fertilized with sperm with matching sex allele(s) so that they become homozygous at the sex locus (sl-CSD, Figure 1) or the sex loci (ml-CSD). In the majority of species diploid males are developmentally unviable or effectively sterile [15,16]. Because sterile diploid males are produced at the expense of fertile females, the fitness consequences of mating a partner with a matching sex allele may be severe.

## Results and discussion

**Figure 2:**
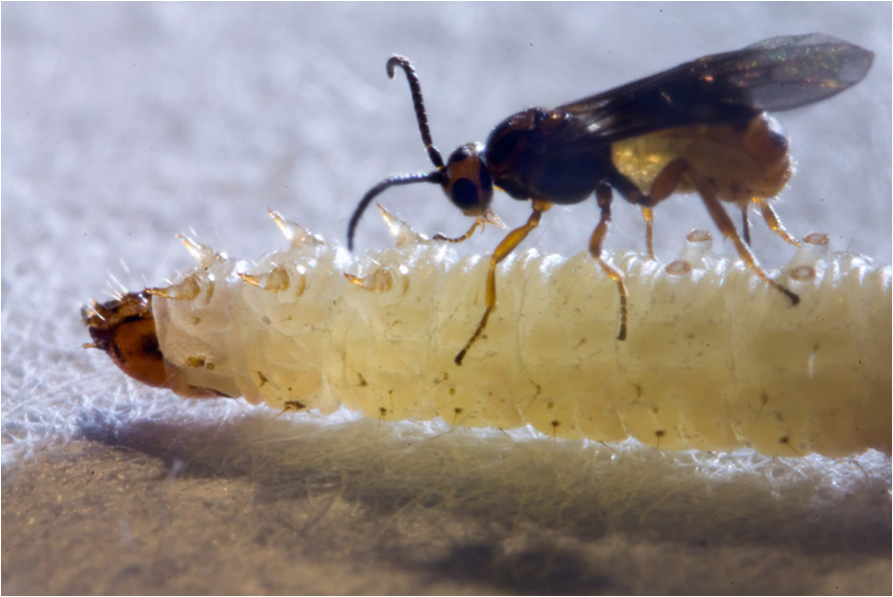
***Bracon brevicornis *habitus.** A female with inserted ovipositor sitting on a paralyzed host (photo: Nils Linek).

The parasitic wasp *Bracon* (*Habrobracon*) *brevicornis* Wesmael (Hymenoptera: Braconidae) (Figure 2), provides an excellent system to evaluate the hypothesis that genetic incompatibility drives mate choice because: a) parental care or resource transfer are absent and benefits of mate choice are purely genetic, b) females usually mate only once [Weeda & Thiel, unpublished observation], and c) genetic incompatibility leads to directly measurable fitness consequences via diploid male production [17,18].

**Figure 3:**
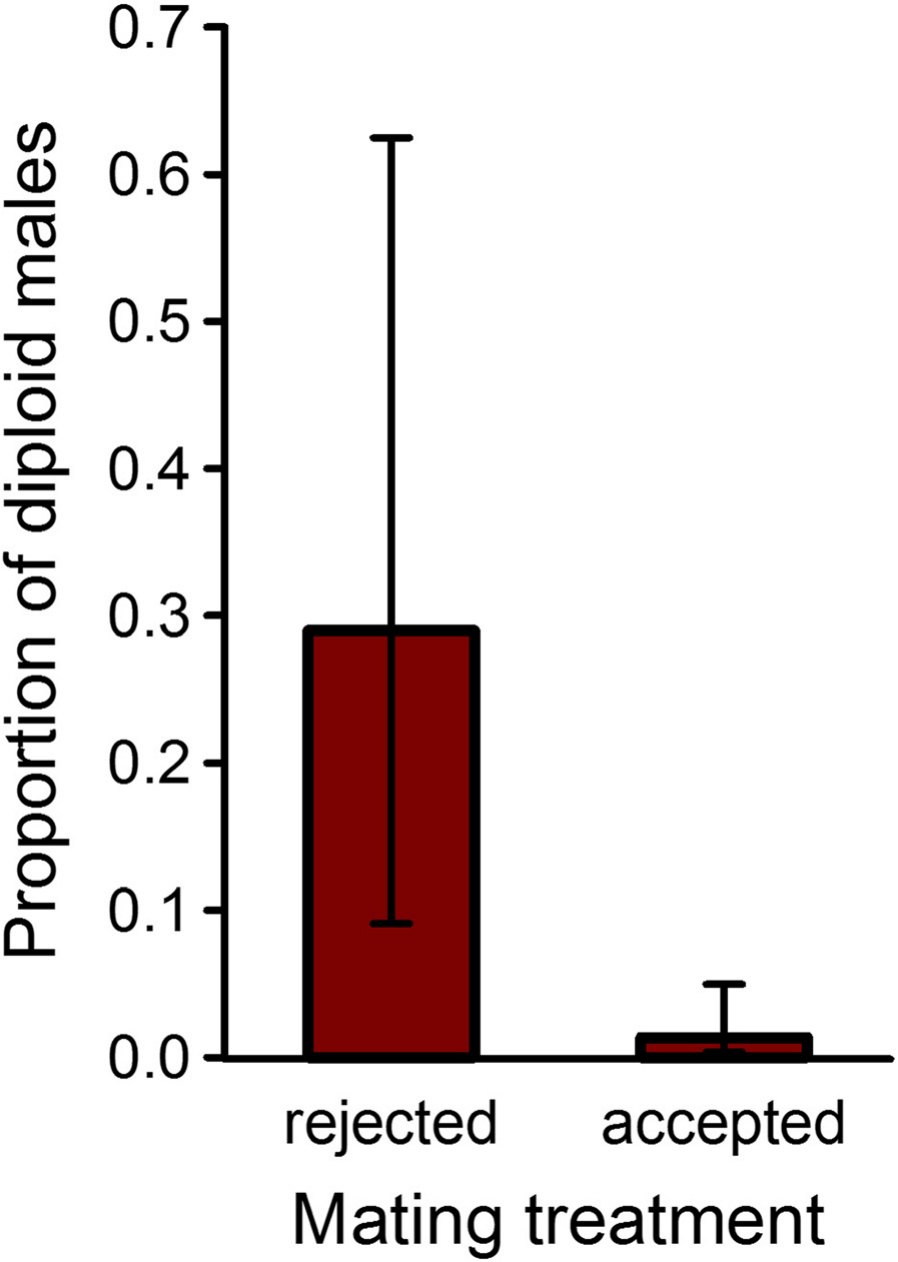
**Mate choice consequences.** The proportion (± SE) of all diploid offspring produced that is male and thus, costly (***P < 0.001).

We used two populations that we assume to partially overlap in the sex alleles present. Individual females of one population were offered a male from the other population and vice versa, and a female could either accept this male (“accepted” mating) or reject it. If rejection occurred, we got the female to accept a second mating attempt of the same male by cooling her on ice (“rejected” mating). From “rejected” matings, a significantly higher proportion of fertilized eggs developed as diploid sons, at the expense of daughters (Χ^2^_df=1, n=20_ = 13.1, *P* < 0.001; error distribution (ED) = binomial, Figure 3). Genetic matching (Table 1) occurred significantly more often in “rejected” than in “accepted” matings (Fisher’s exact test, one-sided *P* = 0.009). However, since not all “rejected” matings have been matched, the females may have had additional mate choice criteria. When looking for alternative explanations for the increased diploid male production in “rejected” matings, we found that neither female fecundity (Χ^2^_1,20_ = 0.007, *P* = 0.93, Table 1) nor offspring mortality (Χ^2^_1,20_ = 1.02, *P* = 0.31) differed significantly between wasps of the different mating regimes.

**Table 1:** **Numbers of eggs produced, haploid male (HM), diploid male (DM), female (F), undetermined male (UM) offspring, and the probability that a matched mating by chance did not result in diploid male production (matching probability, calculated as 0.5**^**(n diploid offspring)**^**)**

**Female**	**Mating**	**Eggs**	**HM**	**DM**	**F**	**UM**	**Matching probability**
1	Accepted	17	0	0	4	0	0.0625
2	Accepted	73	8	0	25	4	<0.0001
3	Accepted	10	0	0	10	0	0.0010
4	Accepted	18	3	0	8	0	0.0039
5	Accepted	69	5	0	55	0	<0.0001
6	Accepted	33	20	2	4	4	Matched
7	Accepted	30	4	0	11	0	0.0005
8	Accepted	37	1	0	7	0	0.0078
9	Accepted	31	10	0	9	0	0.0020
10	Accepted	38	0	0	7	0	0.0078
11	Accepted	33	1	0	7	0	0.0078
12	Rejected	45	4	7	11	0	Matched
13	Rejected	22	4	4	5	2	Matched
14	Rejected	27	5	6	7	0	Matched
15	Rejected	56	20	6	11	5	Matched
16	Rejected	52	21	2	10	5	Matched
17	Rejected	23	13	0	3	0	0.1250
18	Rejected	31	9	0	8	0	0.0039
19	Rejected	34	8	0	13	0	0.0001
20	Rejected	34	3	6	8	0	Matched
21	Control	10	1	2	1	0	Matched
22	Control	38	11	5	3	1	Matched
23	Control	51	9	0	1	0	Matched
24	Control	7	2	1	2	2	Matched
25	Control	26	1	0	3	2	Matched
26	Control	34	9	6	6	0	Matched
27	Control	41	2	7	11	1	Matched
28	Control	54	25	0	1	1	Matched
29	Control	18	9	0	3	0	Matched

Matings are considered matched if DM were produced and in all replicates of the control group (see above). Wasps 1 and 17 were omitted from statistical comparison of matched matings (Fisher test), since the matching probability > 0.05 did not allow for unambiguous classification. Including these data points either as matched or unmatched does not change the conclusions from the Fisher test.

Even though only females from “rejected” mating had been cooled for a short time, an effect of cooling on the proportion of diploid males produced by females with a matched “rejected” mating is unlikely to have occurred. 1) Cooling occurred at a point in time that did neither correlate with egg production, nor sperm storage, nor fertilization. 2) Control females that were mated without cooling to one of their own sons, and thus had a guaranteed matched mating, produced equal proportions of diploid males (Χ^2^_1,15_ = 0.14, *P* = 0.71, Table 1).

Females thus showed the ability to decrease the production of diploid males to approximately 1/4 of what was to be expected at random mating in our experiment. Since diploid sons are produced at the expense of fertile daughters and are effectively sterile in this species [Thiel & Weeda, unpublished observation], this clearly demonstrates the selective advantage of mate choice driven by genetic incompatibility.

## Conclusions

Our results show that in an insect with sl-CSD, recognition of specific alleles can occur even among unrelated mating partners. CSD thus facilitates outcrossing analogous to the self-incompatibility (SI) system found in plants [12] and the MHC system in vertebrates [18]. Yet, fitness consequences of allelic matching are more severe under CSD, compared to MHC or SI, because homozygosity at the CSD-locus invariably increases post-zygotic mortality and the production of infertile offspring [19]. Kin recognition by olfactory cues has been described in a few insect species (e.g. [20-22]) and inbreeding avoidance is certainly an important measure in reducing the probability with which a matched mating is likely to occur [16]. In bottlenecked populations however, even unrelated individuals become likely to share a sex determining allele. As a consequence, a rapid decline of effective population size and a high probability for extinction have been predicted from theoretical models [23,24]. Female choice based on ‘genetic compatibility’, as described in our study, can thus be considered as an important mechanism for increasing population survival as well as individual wasp fitness.

## Materials and methods

We used specimens from two laboratory populations of the gregarious, larval ectoparasitoid *Bracon* (*Habrobracon*) *brevicornis* Wesmael (Hymenoptera: Braconidae) established from collections in two different years (2006 and 2008) from the same field site near Leipzig, Germany. At the time of the experiment, the two populations had been separated for approximately 60 generations and their respective members were thus likely to overlap in the sex determining alleles present, but did not represent close kinships. Larvae of *Ephestia kuehniella* Zeller (Lepidoptera: Pyralidae) served as hosts. Experiments and rearing took place at 25°C, 55% r.h. and 16:8 h light:dark.

We worked with 14-18-day-old virgin females, which had previously parasitized one host. To avoid interference with kin-based mate choice, individual females of one population were placed with a male from the other population, and vice versa, in an empty Petri dish (ø 3.5 cm). A female could either accept the courting male, resulting in copulation usually within five minutes (“accepted” mating), or reject it by bending down the abdomen, kicking with the hind legs, or running away upon contact. If rejection occurred, the female was carefully transferred into a 250 μl plastic tube and placed on ice for eight minutes, until she stopped moving. The non-moving female was transferred back into the Petri dish with the same male she had at first rejected. The male usually approached her immediately and copulation took place before she regained full movement (“rejected” mating; method adapted from [25]). To check for a possible effect of cooling, we used a control group of females that were mated without cooling to one of their own sons, which they had produced as virgins. These females were of the same age than experimental females and had also had one oviposition experience before being mated. Because haploid sons inherit one of their mother’s sex alleles, mother-son matings are by definition matched in terms of sex alleles and should thus result in approximately half of the diploid offspring becoming homozygous at the sex determining locus (diploid males). Interestingly, about 1/2 of those females confronted with the own son mated without hesitation (and could thus be used as a control for effects of cooling), despite the allelic matching. Mating the own son is not contradictory to the idea of avoiding allelic matching if we consider the idea of mate acceptance changing with experience (i.e. [26,27]): if a son is the only potential mate a female encounters, it may well be that there are no conspecifics around and that chances for meeting an unrelated male are really low.

**Figure 4:**
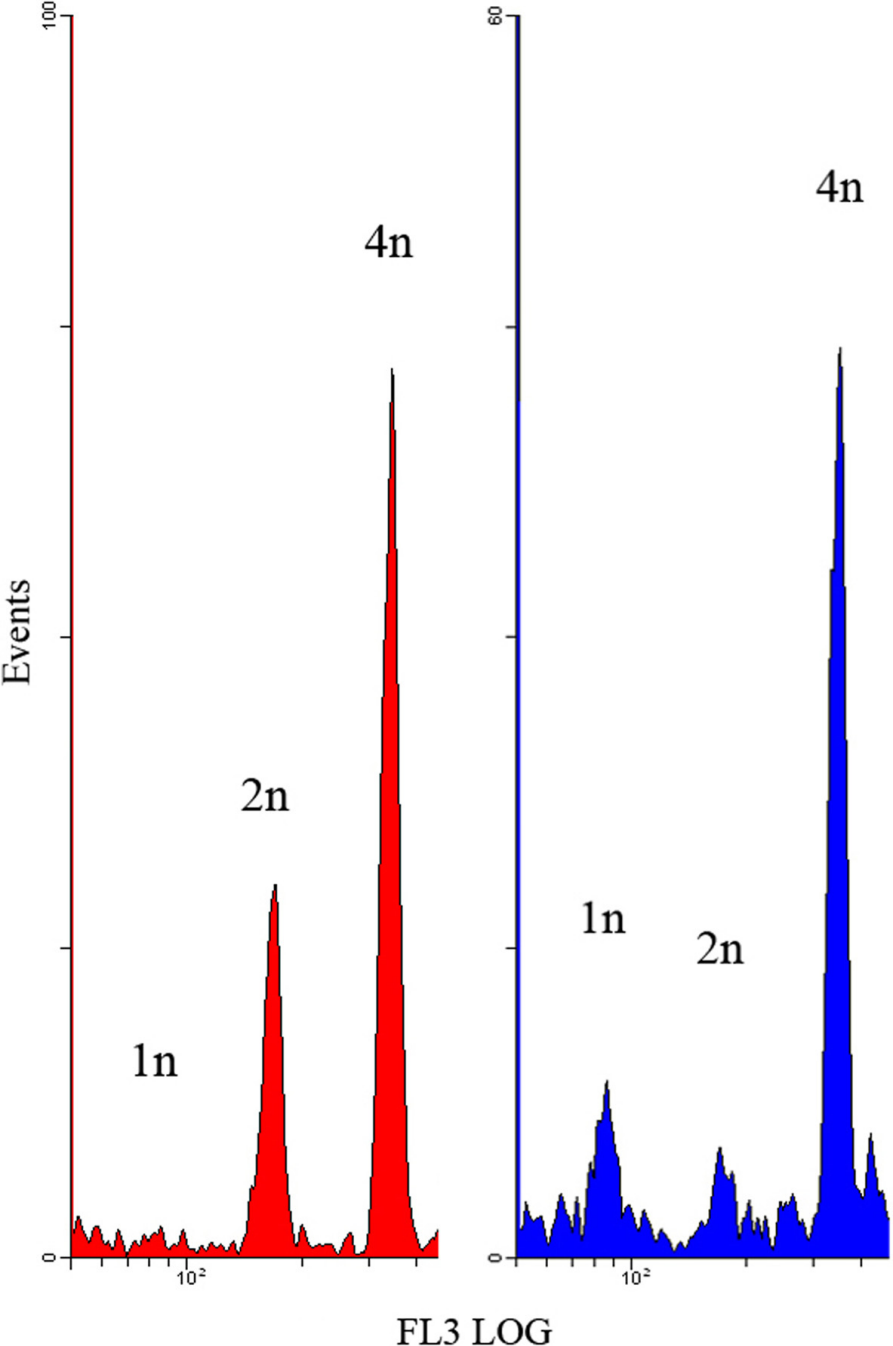
**Flow cytometry reference histograms.** Since DNA duplication is common in Hymenoptera, a haploid individual (right side) is recognized by the presence of a haploid peak, in addition to diploid and tetraploid peaks. A diploid individual is defined by the absence of a haploid peak, while at the same time, diploid and tetraploid peaks are present (left side).

After mating, each female parasitized a total of five host larvae within three days. Host larvae were immediately examined after removing the female to determine the number of eggs laid. After 10 days, we examined developing offspring daily; emerging wasps were sexed and counted. Ploidy levels of male offspring were determined using flow cytometry: individual wasp heads were homogenized in Galbraith buffer (21 mM MgCl_2_, 30 mM trisodium citrate dihydrate, 20 mM MOPS, 0.1% Triton X-100, 1 mg/l RNase [28]), filtered (40 μm), stained for 10 min with 15 μl propidium iodide (1.25 mg/ml) and loaded on a Coulter Epics XL-MCL flow cytometer (Beckman Coulter, Miami, FL, USA). We used an excitation wave length of 488 nm and a band pass filter of 585 nm to detect propidium iodide fluorescence. Samples were measured in an FSlog/FL2-log and FSlog/FL3-log gated region until 2500–3000 counts had been achieved, using *Expo 32 ADC XL 4 Color* (Beckman Coulter, USA). A threshold was applied to exclude very small debris. Known diploids (females) and haploids (males produced by virgin mothers) provided the reference histograms (Figure 4) used for assigning ploidy level to the male offspring produced. Males for which we could not determine ploidy level (Table 1), e.g. because either no definite haploid peak appeared or because the absence of a haploid peak could not be verified, were used only in the analysis of offspring survival.

Generalized linear models (GLMs, [29]) were fitted to the data, unless stated otherwise, using statistical procedures in “R 2.15.2” [30], with package “car” [31]. For analysing the proportions of fertilized eggs developing as diploid males, we used the “cbind” command to account for the different numbers of offspring produced by each female. The error distribution in this test was quasibinomial, with a logit link function. Female fecundity and offspring mortality were analysed with quasipoisson error distributions and log link functions.

Our results show that female choice can reduce the probability of a matched mating to approximately 1/4 of what was to be expected at random mating in our experiment. This calculation is based on those 18 couples for which we could define the matching status with their randomly assigned partners (Table 1): 0.4 of these matings had been matched. Among females that had an “accepted” mating, only a proportion of 0.1 was matched.

## Abbreviations

CSD: Complementary sex determination; F1: First offspring generation; GLM: Generalized linear model; MHC: Major histocompatibility complex; MOPS: 3-(N-morpholino) propanesulfonic acid; P: Parental generation; SI: Self-incompatibility

## Competing interests

The authors declare that they have no competing interests.

## Authors’ contributions

AT initiated and supervised the project, ACW performed the experiments, JGdB directed the flow cytometry. AT and TSH performed the statistics. AT wrote the first draft and all authors contributed to interpreting results, and improvement of the paper. All authors read and approved the final manuscript.
